# Regulation of Silk Genes by Hox and Homeodomain Proteins in the Terminal Differentiated Silk Gland of the Silkworm *Bombyx mori*

**DOI:** 10.3390/jdb4020019

**Published:** 2016-05-25

**Authors:** Shigeharu Takiya, Takuya Tsubota, Mai Kimoto

**Affiliations:** 1 Shigeharu Takiya, Division of Biological Sciences and Center for Genome Dynamics, Faculty of Science, Hokkaido University, North 10, West 8, Kita-ku, Sapporo, Hokkaido 060-0810, Japan; 2 Graduate School of Life Science, Hokkaido University, North 10, West 8, Kita-ku, Sapporo 060-0810, Japan; kimotomai870310@yahoo.co.jp; 3 Transgenic Silkworm Research Unit, Institute of Agrobiological Sciences, National Agriculture and Food Research Organization, 1-2 Owashi, Tsukuba, Ibaraki 305-8634, Japan; tsubota@affrc.go.jp

**Keywords:** Antp, *Bombyx mori*, fibroin, homeodomain, *Hox* genes, promoter element, sericin, silk gland, silkworm

## Abstract

The silk gland of the silkworm *Bombyx mori* is a long tubular organ that is divided into several subparts along its anteroposterior (AP) axis. As a trait of terminal differentiation of the silk gland, several silk protein genes are expressed with unique regional specificities. Most of the *Hox* and some of the homeobox genes are also expressed in the differentiated silk gland with regional specificities. The expression patterns of *Hox* genes in the silk gland roughly correspond to those in embryogenesis showing “colinearity”. The central Hox class protein Antennapedia (Antp) directly regulates the expression of several middle silk gland–specific silk genes, whereas the Lin-1/Isl-1/Mec3 (LIM)-homeodomain transcriptional factor Arrowhead (Awh) regulates the expression of posterior silk gland–specific genes for silk fiber proteins. We summarize our results and discuss the usefulness of the silk gland of *Bombyx mori* for analyzing the function of *Hox* genes. Further analyses of the regulatory mechanisms underlying the region-specific expression of silk genes will provide novel insights into the molecular bases for target-gene selection and regulation by Hox and homeodomain proteins.

## 1. Introduction

The pioneering genetic studies on homeotic mutations in the silkworm *Bombyx mori*, which change the identity of the larval thoracic segments and abdominal segments, were mostly carried out in the first half of the 20th century [1,2,3] and therein. More than 30 homeotic mutations in Nc and E loci have been described in these studies. *Hox* genes responsible for some of these homeotic mutations, and those corresponding to the genes in the Antennapedia (Antp) complex and bithorax complex of *Drosophila*, were subsequently mapped in the *Bombyx* genome structure [4,5,6,7,8,9,10,11].

*Hox* genes are well-known master regulators for determining regional identities along the anteroposterior (AP) axis in most multicellular animals [12,13,14,15]. *Hox* genes encode transcriptional factors, are expressed in different but often overlapping regions along the AP axis during embryogenesis, and regulate different sets of target genes, leading to a different identity of each region in animal bodies. However, the 60-amino-acid-long DNA-binding domains near the C-terminus of Hox proteins, termed homeodomains, are highly conserved and commonly bind to similar adenine (A) and thymine (T)-rich DNA stretches containing the ATTA core, at least *in vitro* [16,17,18,19,20]. This discrepancy is referred to as the “Hox paradox” [21,22,23]. The coordination of Hox proteins with another class of homeodomain proteins, Extradenticle (Exd)/Pbx and Homothorax (Hth)/Meis, increases their DNA-binding specificities [24,25,26,27,28,29,30]. However, these complexes still retain the ability to bind to divergent sequence elements [21,31]. Although genome-wide approaches using microarray or chromatin immunoprecipitation (ChIP) technologies have identified large numbers of *Hox* downstream gene candidates [23,32,33,34,35], the molecular mechanisms by which Hox proteins select and regulate these candidates have not been analyzed in detail. Furthermore, the mechanisms of those leading to functional differences in a Hox protein on target genes also have not been elucidated. Hox proteins can sometimes activate a set of target genes and otherwise repress another set of target genes. Various mechanisms including cofactor-mediated and cofactor-independent DNA-binding steps in addition to DNA-binding-independent steps appear to be involved in the functional regulation of Hox proteins [28,36,37,38,39,40,41,42,43,44,45]. The identification of more target genes and their regulatory elements that are actually recognized by Hox proteins will be useful for understanding more clearly the molecular bases underlying the *in vivo* functions of Hox proteins. We previously reported that the regional specific expression of silk protein genes in the fully differentiated silk gland of the silkworm *Bombyx mori* was directly regulated by several homeodomain-containing transcriptional factors including the Hox protein Antp, and identified their regulatory elements recognized by these homeodomain proteins [46,47,48,49,50,51].

**Figure 1:**
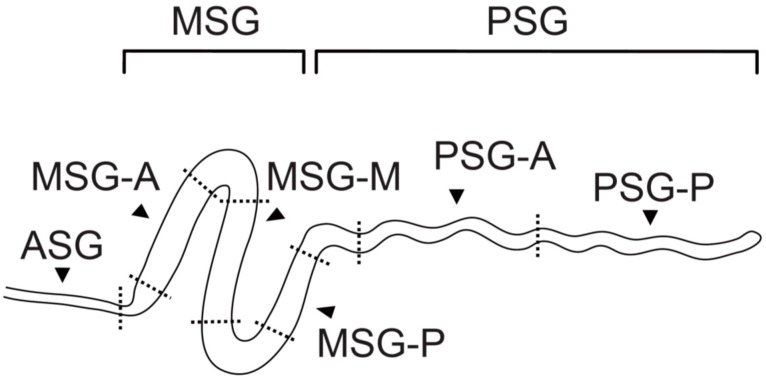
Schematic of the silk gland of *Bombyx mori*. ASG; anterior silk gland, MSG; middle silk gland, MSG-A; anterior portion of the MSG, MSG-M; middle portion of the MSG, MSG-P; posterior portion of the MSG, PSG; posterior silk gland, PSG-A; anterior half of the PSG; PSG-P; posterior half of the PSG. In order to avoid contamination of the neighbor regions, each portion was prepared as shown in this figure. The structural border is not observed in the PSG, and so the long tubular tissue was divided simply into the anterior and posterior halves.

Larvae of the silkworm *Bombyx mori* possess a pair of silk glands. The silk gland is a long tubular organ consisting of a single cell layer of approximately 1000 substantially large, polyploidy cells. Silk gland placodes are induced in the ectoderm of the labial segment by *Sex comb reduced* (*Scr*) during embryogenesis, and the expression of *Scr* disappears from the invaginating silk gland with the *fork head* (*fkh*) and *POU-M1* genes being expressed there instead [52,53,54,55]. The expression of the *fibroin-heavy-chain* gene (*fibH*) is detectable immediately after the completion of silk gland development at around stage 25 of embryogenesis [56]. Silk glands in the silkworm extend from a spinneret on the head to the seventh abdominal segment, and are divided into several subparts along the AP axis (Figure 1) [57,58,59,60,61,62]. Silk fiber proteins are produced in the posterior silk gland (PSG), secreted into the lumen, and then transferred to the middle silk gland (MSG). Several glue proteins are produced in the MSG, secreted into the lumen, and then coat silk fiber proteins. Silk proteins are transferred forward through the cuticle-lined anterior part of the silk gland (ASG), and are spun as silk threads from the spinneret. The MSG is further divided into three subparts: the anterior (MSG-A), middle (MSG-M) and posterior (MSG-P) portions, based on natural turns and their functions [46,47,51,55,57,59,62,63].

Although the silkworms produce a large amount of silk proteins at the end of the last instar to make cocoons, a certain amount of silk proteins is produced constantly from the first instar, except during the molting stages, possibly to keep the larvae stable on mulberry leaves and other substrata. Therefore, the expression of silk protein genes is repeatedly turned on and off in a manner depending on the larval molting cycles [56,58,64,65,66,67]. The mechanisms of this stage-specific expression involve temporal-specific regulation of the activity of Hox and homeodomain-containing transcriptional factors by the insect hormones ecdysone and juvenile hormone, regardless of whether directly or indirectly. We herein describe and discuss the roles of Hox and other homeodomain-containing transcriptional factors in regulating the region-specific expression of silk genes in the terminally differentiated silk gland at the last instar.

## 2. Region-Specific Expression of Silk Genes in the Silk Gland

The *fibroin-heavy-chain* (*fibH*), *fibroin-light-chain* (*fibL*) and *fibrohexamerin* (*fhx*) genes for silk fiber proteins are expressed specifically in PSG cells (Table 1) [64,67,68,69,70]. Silk fiber proteins are assembled into a large elementary unit consisting of Fib-H, Fib-L and Fhx in a 6:6:1 molecular ratio [71], and are then secreted into the lumen. In contrast, the sericin-1 (*ser1*), sericin-2 (*ser2*) and sericin-3 (*ser3*) genes for glue proteins are expressed in MSG cells with sublocal specificities (Table 1) [46,47,51,55,57,59,62,63]. *ser1* is expressed in the MSG-M and MSG-P at the last instar, *ser2* is mainly expressed in the MSG-A, and *ser3* is expressed in the MSG-A and MSG-M. *ser1* mRNAs are differentially spliced depending on the larval stages and the subparts of the MSG, and several isotypes of Ser1 proteins are produced [47,57,72]. *ser2* mRNAs are also differentially spliced [57,62]. In addition to these silk genes, Tsubota *et al.* [51] recently found that other silk genes, *fibrohexamerin-like 4* and *5* (*fhxh4* and *5*), are specifically expressed in the MSG-M and MSG-P as *ser1*. Fhxh4 and Fhxh5 were previously identified as abundant proteins in cocoons [73]. Thus, the expression of silk protein genes is regulated strictly in region-specific manners in the silk gland of *Bombyx mori*.

## 3. Region-Specific Expression of *Hox* and Several Homeobox Genes in the Fully Differentiated Silk Gland

Similar to the silk protein genes, *Hox* and several homeobox genes are known to be expressed with sublocal specificities along the AP axis of the silk gland (Table 1) [6,46,47,55,74,75,76]. The anterior *Hox* genes *labial* (*lab*) and *Scr* are mainly expressed in the ASG, whereas the expression of central *Hox* genes such as *Antp*, *Ultrabithorax* (*Ubx*) and *abdominal-A* (*abd-A*) is absent in the ASG [47]. *Antp* was previously found to be expressed in the MSG, and *abd-A* was expressed in the MSG and PSG. The expression level of *Ubx* was very low, at least at the last instar, but the RT-PCR products were barely detected in the MSG-P and PSG. The posterior *Hox* gene *Abdominal-B* (*Abd-B*) was shown to be expressed in the ASG and posterior half of the PSG [47]. Although we have not yet detected the structural border within the PSG, it may be divided into subparts with different functions. The segment polarity gene *engrailed* (*en*) and its paralogue *invected* (*in*) are strongly expressed in the ASG and posterior portions of the MSG [47,74,76]. The Pit/Oct/Unc-86 (POU)-homeodomain protein gene *POU-M1* is also strongly expressed in the ASG and MSG-A [46,54]. The *Arrowhead* gene (*Awh*), which codes a transcriptional factor belonging to the Lin-1/Isl-1/Mec3 (LIM)-homeodomain family, was shown to be expressed specifically in the PSG [48,49]. Expression of the maternal homeobox gene in *Drosophila* embryogenesis, caudal (cad), was detected in ASG and MSG. These *Hox* and homeobox genes have been expected to serve in the regional specificity of the silk gland and the region-specific expression of silk protein genes.

## 4. Antp Regulates the MSG-Specific Expression of *ser1*

The expression of *ser1* is restricted to the MSG-M and MSG-P in the last instar [46,47,55]. We attempted to identify the promoter elements responsible for the region-specific expression of *ser1*. By using a gene gun, reporter constructs containing the *ser1* promoter were introduced into silk glands dissected out from the last instar larvae, and silk glands were then transplanted into other silkworms at the same stage. After being cultivated for one or two days, the transplanted silk glands were removed from the recipient larvae, and the promoter activity of each construct was assessed. We found that the −70 region of the *ser1* promoter (the transcription start site is denoted +1) was essential for its MSG-specific expression [50]. A protein factor or factor complex binding to the −70 element was detected in extracts prepared from the intermolt MSG-P using the electrophoretic mobility sift assay (EMSA), but not in extracts from the PSG or MSG-P at the molting stage, which is consistent with the spatio-temporal specificity of *ser1* expression. This factor was designated as the MSG-intermolt-specific complex (MIC). The MIC also bound regions further upstream (−1350, −320 and −180) of the *ser1* promoter, and these MIC-binding elements were found to commonly contain the ATTA-core AT-rich sequences recognized by homeodomain proteins (Table 2) [50].

Since *Antp*, *en* and *in* are specifically expressed in the MSG [47,74,76], we prepared recombinant proteins of Antp and En, and determined whether these proteins bind to the −70 element. Although both proteins bound to the −70 element of the *ser1* promoter, Antp but not En showed a similar DNA-binding specificity to that of MIC [47]. The apparent molecular size of the Antp-probe DNA complex detected with EMSA was markedly smaller than that of the MIC-probe complex. Hox proteins are known to make a complex with the TALE class homeodomain proteins extradenticle (Exd) and homothorax (Hth) via the conserved hexapeptide motif that resides on the N-terminal side of the homeodomain of Hox proteins [28,29]. The addition of recombinant Exd and Hth proteins together with Antp into EMSA reactions led to the formation of a MIC-like complex with a similar size to that of MIC. Using antibodies specific to Antp, Exd or Hth in EMSA, we confirmed that the MIC in MSG-P extracts included these Hox and homeodomain proteins [47], though the −70 element of *ser1* does not contain apparent binding sequences for Exd or Hth, except the ATTA core commonly recognized by homeodomain proteins. Although a hexapeptide-independent interaction between Exd and several Hox proteins has been reported [88,89], the YPWM motif of Antp appeared to be necessary for the formation of MIC with the cofactors Exd and Hth on the −70 element of the *ser1* promoter.

When the recombinant Antp protein was added to extracts from the PSG, the formation of a MIC-like complex was observed via EMSA. In addition, the genes for Exd and Hth were expressed in the PSG as in the MSG. We investigated whether the misexpression of Antp in the PSG induces ectopic expression of *ser1*. The expression of *ser1* was induced in the PSG of transgenic silkworms expressing *Antp*. When the binding of MIC to the −70 element was disturbed by two-nucleotide mutations, the promoter activity of *ser1* was mostly lost [50]. Thus, the central Hox protein Antp is an essential activator of *ser1* expressed specifically in the middle portions (MSG-M and MSG-P) of the fully differentiated silk gland of normal silkworms [47], and the activation function of Antp is achieved through binding to the −70 element of *ser1* promoter.

## 5. Antp Activates Other MSG-Specific Silk Genes

Tsubota *et al.* [51] recently found the novel Antp-induced silk genes *fhxh4* and *fhxh5*. *fhxh4* and *fhxh5* are specifically expressed in the MSG-M and MSG-P as *ser1* in wild-type silkworms, whereas the ectopic expression of these genes was induced in the PSG when *Antp* was misexpressed in transgenic silkworm lines. The ectopic expression of *ser3* in the PSG induced by Antp was also observed. Consensus MIC-binding sequences have been identified in the upstream regions of these genes (Table 2), and the binding of MIC to these elements was confirmed with EMSA, suggesting that these MSG-specific silk genes are direct targets of Antp, similar to *ser1* [51]. *Antp* appears to function as a master regulator for MSG-specific gene expression. The expression of *ser3* is restricted to the MSG-A and MSG-M in wild-type silkworms. mRNAs for *Antp* are detected in the MSG-A, but the Antp protein was not detected with Western blotting, and even with the addition of recombinant Antp into MSG-A extracts, the MIC-like complex was not formed in EMSA [47]. Therefore, the expression of *ser3* in the MSG-A appears to be regulated by an Antp-independent mechanism.

## 6. The LIM-Homeodomain Protein, Arrowhead, Regulates the PSG-Specific Expression of Silk Fiber Protein Genes

Of the PSG-specific silk fiber protein genes, the promoter of *fibH* has been analyzed in detail using cell-free transcription systems prepared from PSG and other tissues of the silkworm [79,90,91,92,93,94,95,96]. The activity of natural DNA of the *fibH* promoter obtained from the PSG without cloning technology has also been assessed in cell-free systems [97]. Regulatory elements bound by various transcription factors such as Fork head/silk gland factor-1 (Fkh/SGF-1), silk gland factor-2 (SGF-2), POU-M1/SGF-3 and fibroin-modulator-binding protein-1 (FMBP-1) were detected (Table 2) [78,79,80,84,92,98]. In these studies, the SGF-2-binding elements present from −238 to −73 were found to be essential for efficient transcription of *fibH* in PSG extracts [78,91,94,95]. Furthermore, Shimizu *et al.* [77] demonstrated that the combination of two far-upstream enhancer elements with promoter elements is indispensable for the full activation of *fibH in vivo* using a gene gun system, and an SGF-2-binding sequence at approximately −1620 was a key element in the far-upstream enhancer.

The LIM-homeodomain protein Arrowhead (Awh) was found to be an essential factor regulating PSG-specific expression of *fibH*, *fibL* and *fhx*. SGF-2 was purified from PSG extracts by Ohno *et al.* [49], and it was shown to be composed of Awh, LIM-domain binding protein (Ldb) and a member of a single-stranded DNA-binding protein family (LIM-homeodomain and Ldb complex associated factor; Lcaf). In transgenic *Drosophila*, a reporter gene having *Bombyx* *fibH* or the *fhx* promoter are expressed in a restricted region around the imaginal ring of the salivary gland, which resembles the expression region of the *Drosophila* *Awh* gene [99,100,101]. The salivary gland of *Drosophila* develops from the labial segment under the regulation of *Scr*, as does the *Bombyx* silk gland; however, the *Bombyx* salivary gland is derived from the mandibular segment. In *Bombyx mori*, *Awh* is expressed in a strictly specific manner in the PSG, but not in the salivary gland in the last instar, whereas *Ldb* and *Lcaf* are expressed in most tissues [48,49]. The misexpression of *Awh* in transgenic silkworms has been shown to induce the ectopic expression of *fibH*, *fibL*, and *fhx* in the MSG, and SGF-2-binding elements have also been detected around the promoter of *fibL* and *fhx* (Table 2). We previously reported that the core promoter of *fhx* itself is an SGF-2-binding element, while the core promoter of *fibL* has the ability to function as a binding element for both Fkh and FMBP-1. The core promoter of *fhx* and *fibL* may act as a tissue-specific regulatory element.

*Ldb* and *Lcaf* are expressed in most tissues in last-instar larvae, and the misexpression of *Awh* was induced in the whole body from a heat-shock promoter in transgenic silkworms. However, the ectopic expression of *fibH* was induced only in the MSG. Under the same conditions, ectopic expression of *fibL* and *fhx* was induced in the ASG in addition to the MSG [48]. The findings suggest that SGF-2 directly regulates the expression of these PSG-specific silk genes, and that coordination with other factors such as Bmsage, which is a silk gland–specific basic helix-loop-helix transcription factor interacting with Fkh, may be necessary [102]. The coordination of these transcription factors will be interpreted by the context (*i.e.*, structure) of each promoter. The expressions of *fibH* and *ser1* were not induced in the ASG under the misexpression of *Awh* and *Antp*, respectively, and the expression level of *fibH* in the MSG-P induced by the misexpression of *Awh* was very low [47,48]. *POU-M1*, *en* and *in*, which are efficiently expressed in these portions, may exert a suppressive function on these genes [46,55,74,76,98,103]. A typical POU-M1-binding element was detected in the promoter of *fibH* and *ser-1* (Table 2), but not around the promoter of *fibL* and *fhx*. In *Drosophila*, En has the ability to recruit the corepressor Groucho and repress target genes [104,105,106]. For example, En suppresses the expression of *fkh* under coordination with Abd-A in *Drosophila*.

## 7. Perspective

We have demonstrated that the homeodomain proteins Antp and Awh are involved in regulating the region-specific expression of silk genes in the silk gland of *Bombyx mori* by direct binding to the regulatory elements of the silk genes [47,48,49,50,51]. The region-specific regulatory functions of Antp and Awh on the silk genes are achieved through region-specific expression of *Antp* and *Awh*. Therefore, it is important to analyze the upstream regulatory mechanisms responsible for the region-specific expression of *Antp* and *Awh*. In the silk gland, other *Hox* and homeobox genes are also expressed with unique regional specificities along the AP axis, and the homeodomain-containing transcriptional factors encoded by these genes may be involved in the cascades regulating the regional specific expression of silk genes. A large number of ATTA core AT-rich elements consist of the consensus sequences recognized by homeodomain proteins in and around the silk genes’ promoters [50,51,78,107,108]. Several AT-rich stretches have also been found around the promoter of *Awh* (Takiya, unpublished results). Therefore, the silk gland of *Bombyx mori* is an appropriate system for analyzing how Hox and other homeodomain proteins select and regulate their specific target genes *in vivo*, in spite of the relative lack of DNA-binding specificity *in vitro*. This will provide an insight into the mechanisms by which *Hox* genes determine regional identities along the AP axis of animal bodies.

The expression of *Antp* mRNA was detectable in the entire MSG, whereas the Antp protein was only found in the MSG-M and MSG-P and was scarcely detectable in the MSG-A with Western blotting [47]. These findings suggest that post-transcriptional regulatory mechanisms also restrict the function of *Antp* in the silk gland. *Antp* mRNA of *Drosophila* has an internal ribosomal entry site (IRES), and the activity of *Antp* is regulated not only at the step of transcription, but also at the step of translation. [109]. The translational regulation of some *Hox* mRNAs is necessary for the normal development of vertebrates [110,111]. Therefore, it is possible that other *Hox* genes expressed in the silk gland are regulated at translational and/or post-translational steps, as is *Antp*.

The expression pattern of *Hox* genes along the AP axis during embryogenesis corresponds to the tandem array on the chromosome and is called “colinearity” [12,112]. Although colinearity is conserved in many animals, the biological significance of maintaining the colinearity during animal evolution remains unclear. Expression levels of *Hox* genes appear to be affected by the distance from one enhancer, and the distance from the remote enhancer determines the chromatin structure affecting the expression level of each *Hox* gene [112,113,114]. When *Hox* genes are expressed in overlapped regions, the more posterior *Hox* genes tend to govern specificity of the regions. The phenomenon is termed “posterior prevalence”. It is not clear at present whether the mechanisms underlying posterior prevalence play a role in the silk gland. However, *Hox* genes are expressed in regionally specific, but partly overlapping patterns that roughly correspond to those during embryogenesis. The mechanisms of posterior prevalence may be involved in the differentiation of each part of the silk gland and also in the region-specific expression of silk protein genes.

The silk gland of *Bombyx mori* is a large and single-cell layered organ, and it is easy to obtain many materials for the preparation of proteins, RNAs and other cell components produced in a region-specific manner. We have the ability to analyze the promoter activities of various constructs transiently in the silk gland and other tissues using a gene gun system [50,77,81,101,115,116]. Technologies to prepare transgenic silkworms and manipulate the genome have been established and are being improved [117,118,119,120,121]. Therefore, the silk gland is an appropriate system to analyze the issues described above both biochemically and genetically. The silkworm *Bombyx mori* in which Mendel’s law was first confirmed in the animal kingdom [122] is promising to provide novel insight into the molecular basis underlying the regulation of animal body plans by *Hox* genes.

## Figures and Tables

**Table 1:** Region-specific expression of the genes for silk proteins, and Hox and some homeodomain-containing transcriptional factors in the silk gland.

Silk Gene	ASG ^(1)^	MSG			PSG		Reference
		Anterior ^(1)^	Middle ^(1)^	Posterior ^(1)^	Anterior ^(1),(2)^	Posterior ^(1),(2)^	
Silk Fiber Protein							
*fibH*	−	−	−	−	+++ ^(3)^	+++	[48]
*fibL*	−	−	−	−	+++	+++	[48]
*fhx*	−	−	−	−	+++	+++	[48]
Glue Protein							
*ser1*	−	−	+++	+++	−	−	[46,47,51]
*ser2*	−	+++	++	−	−	−	[51]
*ser3*	−	+++	+++	−	−	−	[51]
*fhxh4*	−	−	++	+++	−	−	[51]
*fhxh5*	−	−	++	+++	−	−	[51]
Hox Gene							
*lab*	++	+/−	+	+	+	+	[47]
*Scr*	++	+/−	−	−	−	−	[47]
*Antp*	−	++ ^(4)^	++	++	−	−	[47]
*Ubx*	−	−	−	+/−	+/−	+/−	[47]
*abd-A*	−	+	+	+	+	+	[47]
*Abd-B*	+	+/−	−	−	−	+	[47]
Homeobox Gene ^(5)^							
*Awh*	−	−	−	−	++	++	[48]
*En*	+	+/−	+	+	−	−	[47]
*in*	++	−	+	++	−	−	[47]
*Pou-m1*	++	++	+	−	−	−	[46,47]
*cad*	+	+/−	+/−	+/−	−	−	[47]
*exd*	++	++	++	++	++	++	[47]
*hth*	+	+/−	++	++	+	+	[47]

^(1)^ Total RNA prepared from each region of the silk gland at two days or three days (in the reference [51]) of the fifth instar was used for RT-PCR; ^(2)^ The structral border is not observed within the PSG. The long tissue was divided simply into the anterior and posterior halves.; ^(3)^ Results of RT-PCR are summarized. (+++) detectable with less than 25 cycles, (++) detectable with 30 cycles, (+) detectable with 35 cycles, (+/−) barely detectable with more than 35 cycles, and (−) undetectable. Expression levels were not be compared between different genes.; ^(4)^ The *Antp* gene is efficiently transcribed, whereas the Antp protein was not detected in the MSG-A with Western blotting.; ^(5)^ Genes encoding homeodomain-containing proteins except for the *Hox* genes.

**Table 2:** Nucleotide sequence around the regulatory elements of the silk gene promoter.

Gene	Position	Strand	Factor	Sequence	Reference
Silk Fiber Protein					
*fibH*	−1620	−	SGF-2	CTTG CAATTA AGCACTTATTC	[77]
	−200	+	SGF-2	GAT CAATTA AAT CATAATTA ATC ^(3)^	[48,49,78]
	−110	−	SGF-2	GATA CAATTA CATAG AAATTA ATC ^(3)^	[48,49,78]
	−270	−	Fkh ^(1)^	GTAA TATTTAAAGA ACTTA	
	−130	−	Fkh, FMBP-1	ATCT TTTTATTTAACAT AACAA ^(4)^	[79,80]
	−70	+	Fkh	TAGA TGTTTATTCT ATCG	[78]
	−60	−	Fkh ^(2)^	GACG TATTTACTTT CGAT	
	−130	+	POU-M1	TGTT ATGTTAAA TAAA	[78]
	−110	+	POU-M1	TTCT ATGTAATT GTATC	[78]
*fibL*	−230	+	SGF-2 ^(1)^	GGAT CAATTA GATCGCTTTG	
	−50	−	SGF-2	AAGA CAATTA AAA TAAATA TC ^(3)^	[48]
	−50	+	Fkh ^(1)^, FMBP-1 ^(1)^	TTGA TATTTATTTT AATTG ^(4)^	
	−30	−	Fkh ^(1)^, FMBP-1 ^(1)^	CCAC TATTTATATA TAAAA ^(5)^	
*fhx*	−30	+	SGF-2	GGAA CAAT ACTTG TATAATTA ATGTTG ^(6)^	[48,81]
	+100	+	SGF-2 ^(1)^	GGT CAATTA TAACTAC	
	−70	+	Fkh, FMBP-1 ^(1)^	ACGC TATTTATTTA ACGT ^(4)^	[81,82,83]
Glue Protein					
*ser1*	−1350	+	MIC	TAATGC AATTAATATC GTATC	[50]
	−310	+	MIC	AATTCC AATTAATTAT AGTCG	[50]
	−180	+	MIC	GAAATC AATTAATAAC ATAAA	[50]
	−70	+	MIC	GCGAA AATTTATTAC TCTCT	[50]
	−160	−	Fkh ^(1)^	ATTT TGTTTGCCTA TTTTA	
	−120	+	Fkh ^(1)^	AGAA CGTTTGTTGA ACAA	
	−90	+	Fkh	ACAT TGTTTGCACA AATGTT	[84,85,86]
	−200	+	POU-M1	AGCC ATGAATAA ATTAG	[85,86]
	−140	−	POU-M1	CTCT ATGTAAAT GGTTT	[85,86]
*ser3*	−90	+	MIC, Fkh ^(2)^, FMBP-1 ^(1)^	AAAT AATTAATTATTTATTTT ATTG ^(4)^	[51]
	−100	−	Fkh ^(2)^	TAAT TATTTGTTTA ATACAC	
	−30	−	Fkh ^(1)^	CGGC TATTTATACT AATTT ^(7)^	
	−70	−	POU-M1 ^(1)^	CTTT ATGAATAA ACAG	
*fhxh4*	−1660	−	MIC	AAATT GATTTATGAC AGAG	[51]
	−180	−	Fkh ^(1)^	TTTT TGTTTAATTA TTAT	
	−160	−	Fkh ^(1)^	TTTT TGTTTAATTT TTTT	
	−60	+	Fkh ^(1)^	TAAA TGTTTATTTT CTTAT	
	−40	−	Fkh ^(1)^	ACTG TGTTTAAATT ATGTT	
	−30	+	Fkh ^(1)^	TGCT TATTTATATG TAAG ^(8)^	
*fhxh5*	−300	−	MIC	AATGA GATTTATAAT ATTGAT	[51]
	−200	+	Fkh ^(1)^	CTTG TATTTAGATT ATTTA	
	−130	+	Fkh ^(1)^	ATATAA TATTTAATGT AAACG	
	−60	−	Fkh ^(1)^	GTGTAA TATTTGCTGG ATATTA	
	−30	−	Fkh ^(1)^	AAACT AGTTTGTATA ATTCC	

^(1)^ Presumption from consensus sequences; ^(2)^ Takiya, unpublished results; ^(3)^ Paired SGF-2-binding element; ^(4)^ The FMBP-1-binding element “ATNTWTNTA” or its variations overlapped; ^(5)^ The Fkh-binding element and FMBP-1-binding element overlapped with the TATA box; ^(6)^ The incomplete paired SGF-2-binding element overlapped with the TATA box; ^(7)^ The Fkh-binding element overlapped with the TATA box; ^(8)^ The TATA box of *fhxh4* might be a weak Fkh-binding element. Affinity of Fkh to various sequence elements was estimated experimentally in reference [87].

## References

[1] Tazima Y. (1964). The Genetics of the Silkworm.

[2] Suzuki Y. (1994). Genes that are involved in *Bombyx* body plan and silk gene regulation. Int. J. Dev. Biol..

[3] Ueno K., Nagata T., Suzuki Y., Goldsmith M.R., Wilkins A.S. (1995). Roles of homeotic genes in the *Bombyx* body plan. Molecular Model Systems in the Lepidoptera.

[4] Chai C.-L., Zhang Z., Huang F.-F., Wang X.-Y., Yu Q.-Y., Liu B.-B., Tian T., Xia Q.-Y., Lu C., Xiang Z.-H. (2008). A genomewide survey of homeobox genes and identification of novel structure of the Hox cluster in the silkworm *Bombyx mori*. Insect Biochem. Mol. Biol..

[5] Masumoto M., Yaginuma T., Niimi T. (2009). Functional analysis of *Ultrabithorax* in the silkworm, *Bombyx mori* using RNAi. Dev. Genes Evol..

[6] Nagata T., Suzuki Y., Ueno K., Kokubo H., Xu X., Hui C.-C., Hara W., Fukuta M. (1996). Developmental expression of the *Bombyx Antennapedia* homologue and homeotic changes in the Nc mutant. Genes Cells.

[7] Pan M.-H., Wang X.-Y., Chai C.-L., Zhang C.-D., Lu C., Xiang Z.-H. (2009). Identification and function of *abdominal-A* in the silkworm, *Bombyx mori*. Insect Mol. Biol..

[8] The International Silkworm Genome Consortium (2008). The genome of a lepidopteran model insect, the silkworm *Bombyx mori*. Insect Biochem. Mol. Biol..

[9] Tomita S., Kikuchi A. (2009). *Abd-B* suppresses lepidopteran proleg development in posterior abdomen. Dev. Biol..

[10] Ueno K., Hui C.-C., Fukuta M., Suzuki Y. (1992). Molecular analysis of the deletion mutants in the E homeotic complex of the silkworm *Bombyx mori*. Development.

[11] Yasukochi Y., Ashakumary L.A., Wu C., Yoshido A., Nohata J., Mita K., Sahara K. (2004). Organization of the Hox gene cluster of the silkworm, *Bombyx mori*: A split of the Hox cluster in a non-*Drosophila* insect. Dev. Genes Evol..

[12] Lewis E.B. (1978). A gene complex controlling segmentation in *Drosophila*. Nature.

[13] Mann R.S., Carroll S.B. (2002). Moleculae mechanisms of selector gene function and evolution. Curr. Opin. Genet. Dev..

[14] Mann R.S., Morata G. (2000). The developmental and molecular biology of genes that subdivide the body of *Drosophila*. Annu. Rev. Cell Dev. Biol..

[15] McGinnis W., Krumlauf R. (1992). Homeobox genes and axial patterning. Cell.

[16] Berger M.F., Badis G., Gehrke A.R., Talukder S., Philippakis A.A., Penta-Castillo L., Alleyene T.M., Mnaimneh S., Botvinnik O.B., Chan E.T. (2008). Variation in homeodomain DNA binding revealed by high-resolution analysis of sequence preferences. Cell.

[17] Ekker S.C., Jackson D.G., Kessler D.P., Sun B.I., Young K.E., Beachy P.A. (1994). The degree of variation in DNA sequence recognition among four *Drosophila* homeotic proteins. EMBO J..

[18] Gehring W.J., Qian Y.Q., Billeter M., Furukubo-Tokunaga K., Schier A.F., Resendez-Perez D., Affolter M., Otting G., Wuthrich K. (1994). Homeodomain-DNA recognition. Cell.

[19] Mann R.S. (1995). The specificity of homeotic gene function. BioEssays.

[20] Noyes M.B., Christensen R.G., Wakabayashi A., Stormo G.D., Brodsky M.H., Wolfe S.A. (2008). Analysis of homeodomain specificities allows the family-wide prediction of preferred recognition sites. Cell.

[21] Ebner A., Cabernard C., Affolter M., Merabet S. (2005). Recognition of distinct target sites by a unique Labial/Extradenticle/Homothorax complex. Development.

[22] Hayashi S., Scott M.P. (1990). What determines the specificity of action of *Drosophila* homeodomain proteins?. Cell.

[23] Huber S.D., Lohmann I. (2008). Shaping segments: *Hox* gene function in the genomic age. BioEssays.

[24] Chang C.-P., Brocchieri L., Shen W.-F., Largman C., Cleary M.L. (1996). Pbx modulation of Hox homeodomain amino-terminal arms establishes different DNA-binding specificities across the *Hox* locus. Mol. Cell Biol..

[25] Chan S.-K., Jaffe L., Capovilla M., Botas J., Mann R.S. (1994). The DNA binding specificity of Ultrabithorax is modulated by cooperative interactions with extradenticle, another homeoprotein. Cell.

[26] Chan S.-K., Mann R.S. (1996). A structural model for a homeotic protein-extradenticle-DNA complex accounts for the choice of HOX protein in the heterodimer. Proc. Natl. Acad. Sci. USA.

[27] Joshi R., Passner J.M., Rohs R., Jain R., Sosinsky A., Crickmore M.A., Jacob V., Aggarwal A.K., Honig B., Mann R. (2007). Functional specificity of a Hox protein Mediated by the recognition of minor groove structure. Cell.

[28] Mann R.S., Lelli K.M., Joshi R. (2009). Hox specificity: Unique roles for cofactors and collaborators. Curr. Top. Dev. Biol..

[29] Moens C.B., Selleri L. (2006). Hox cofactors in vertebrate development. Dev. Biol..

[30] Slattery M., Riley T., Liu P., Abe N., Gomez-Alcala P., Dror I., Zhou T., Rohs R., Honig B., Bussemaker H.J. (2011). Cofactor binding evokes latent differences in DNA binding specificity between Hox proteins. Cell.

[31] Uhl J.D., Cook T.A., Gebelein B. (2010). Comparing anterior and posterior Hox complex formation reveals guidelines for predicting *cis*-regulatory elements. Dev. Biol..

[32] Agrawal P., Habib F., Yelagandula R., Shashidhara L.S. (2011). Genome-level identification of targets of Hox protein Ultrabitholax in *Drosophila*: Novel mechanisms for target selection. Sci. Rep..

[33] Hersh B.M., Nelson C.E., Stoll S.J., Norton J.E., Albert T.J., Caroll S.B. (2007). The UBX-regulated network in the haltere imaginal disc of *D*. *melanogaster*. Dev. Biol..

[34] Hueber S.D., Bezdan D., Henz S.R., Blank M., Wu H., Lohmann I. (2007). Comparative analysis of Hox downstream genes in *Drosophila*. Development.

[35] Slattery M., Ma L., Negre N., White K.P., Mann R.S. (2011). Genome-wide tissue-specific occupancy of the Hox protein Ultrabithorax and Hox cofactor Homothorax in Drosophila. PLoS ONE.

[36] Galant R., Caroll S.B. (2002). Evolution of a transcriptional repression domain in an insect Hox protein. Nature.

[37] Galant R., Walsh C.M., Caroll S.B. (2002). Hox repression of a target gene: extradenticle-independent, additive action through multiple monomer binding sites. Development.

[38] Krasnow M.A., Saffman E.E., Kornfeld K., Hogness D.S. (1989). Transcriptional activation and repression by *Ultrabithorax* proteins in cultured *Drosohila* cells. Cell.

[39] Li X., Murre C., McGinnis W. (1999). Activity regulation of a Hox protein and a role for the homeodomain in inhibiting transcriptional activation. EMBO J..

[40] Merabet S., Kambris Z., Capovilla M., Berenger H., Pradel J., Graba Y. (2003). The hexapeptide and linker regions of the AbdA Hox protein regulate its activating and repressive functions. Dev. Cell.

[41] Papadopoulos D.K., Resendez-Perez D., Cardenas-Chavez D.L., Villanueva-Segura K., Canales-del-Castillo R., Felix D.A., Funfschilling R., Ghering W.J. (2011). Functional synthetic *Antennapedia* genes and the dual roles of YPWM motif and linker size in transcriptional activation and repression. Proc. Natl. Acad. Sci. USA.

[42] Prince F., Katuyama T., Oshima Y., Plaza S., Resendez-Perez D., Berry M., Kurata S., Ghering W.J. (2008). The YPWM motif links Antennapedia to the basal transcriptional machinery. Development.

[43] Ronshaugen M., McGinnis N., McGinnis W. (2002). Hox protein mutation and macroevolution of the insect body plan. Nature.

[44] Saadaoi M., Merabet S., Litim-Mecheri I., Arbeille E., Sambrani N., Damen W., Brena C., Pradel J., Graba Y. (2011). Selection of distinct Hox-Extradenticle interaction modes fine-tunes Hox protein activity. Proc. Natl. Acad. Sci. USA.

[45] Winslow G.M., Hayashi S., Krasnow M., Hogness D.S., Scott M.P. (1989). Transcriptional activation by the *Antennapedia* and *fushi tarazu* proteins in cultured *Drosophila* cells. Cell.

[46] Kimoto M., Kitagawa T., Kobayashi I., Nakata T., Kuroiwa A., Takiya S. (2012). Inhibition of the binding of MSG-intermolt-specific complex, MIC, to the *sericin-1* gene promoter and *sericin-1* gene expression by POU-M1/SGF3. Dev. Genes Evol..

[47] Kimoto M., Tsubota T., Uchino K., Sezutsu H., Takiya S. (2014). Hox transcription factor Antp regulates *sericin-1* gene expression in the terminal differentiated silk gland of *Bombyx mori*. Dev. Biol..

[48] Kimoto M., Tsubota T., Uchino K., Sezutsu H., Takiya S. (2015). LIM-homeodomain transcription factor Awh is a key component activating all three fibroin genes, *fibH*, *fibL* and *fhx*, in the silk gland of the silkworm, *Bombyx mori*. Insect Biochem. Mol. Biol..

[49] Ohno K., Sawada J., Takiya S., Kimoto M., Matsumoto A., Tsubota T., Uchino K., Hui C.-C., Sezutsu H., Handa H. (2013). Silk gland factor-2, involved in fibroin gene transcription, consists of LIM homeodomain, LIM-interactiong, and single-stranded DNA-binding proteins. J. Biol. Chem..

[50] Takiya S., Inoue H., Kimoto M. (2011). Novel enhancer and promoter elements indispensable for the tissue-specific expression of the *sericin-1* gene of the silkworm *Bombyx mori*. Insect Biochem. Mol. Biol..

[51] Tsubota T., Tomita S., Uchino K., Kimoto M., Takiya S., Kajiwara H., Yamazaki T., Sezutsu H. (2016). A *Hox* gene, *Antennapedia*, regulates expression of multiple major silk protein genes in the silkworm *Bombyx mori*. J. Biol. Chem..

[52] Kokubo H., Takiya S., Mach V., Suzuki Y. (1996). Spatial and temporal expression pattern of *Bombyx fork head*/SGF-1 gene in embryogenesis. Dev. Genes Evol..

[53] Kokubo H., Ueno K., Amanai K., Suzuki Y. (1997). Involvement of the *Bombyx Scr* gene in development of the embryonic silk gland. Dev. Biol..

[54] Kokubo H., Xu P.-X., Xu X., Matsunami K., Suzuki Y. (1997). Spatial and temporal expression pattern of POU-M1/SGF-3 in *Bombyx mori* embryogenesis. Dev. Genes Evol..

[55] Matsunami K., Kokubo H., Ohno K., Suzuki Y. (1998). Expression pattern analysis of SGF-3/POU-M1 in relation to sericin-1 gene expression in the silk gland. Dev. Growth Differ..

[56] Ohta S., Suzuki Y., Hara W., Takiya S., Suzuki T. (1988). Fibroin gene transcription in the embryonic stages of the silkworm, *Bombyx mori*. Dev. Growth Differ..

[57] Couble P., Michaille J.-J., Garel A., Couble M.-L., Prudohmme J.-C. (1987). Developmental switches of sericin mRNA splicing in individual cells of *Bombyx mori* silk gland. Dev. Biol..

[58] Ishikawa E., Suzuki Y. (1985). Tissue-and stage-specific expression of sericin genes in the middle silk gland of *Bombyx mori*. Dev. Growth Differ..

[59] Michaille J.-J., Garel A., Prudhomme J.-C. (1990). Cloning and characterization of the highly polymorphic *Ser2* gene of *Bombyx mori*. Gene.

[60] Obara T., Suzuki Y. (1988). Temporal and spatial control of silk gene transcription analyzed by nuclear run-on assay. Dev. Biol..

[61] Suzuki Y., Takiya S., Suzuki T., Hui C.-C., Matsuno K., Fukuta M., Nagata T., Ueno K., Hagedorn H.H., Hildebrand J.G., Kidwell M.G., Law J.H. (1990). Developmental regulation of silk gene expression in *Bombyx mori*. Molecular Insect Science.

[62] Takasu Y., Yamada H., Tamura T., Sezutsu H., Mita K., Tsubouchi K. (2007). Identification and characterization of a novel sericin gene expressed in the anterior middle silk gland of the silkworm *Bombyx mori*. Insect Biochem. Mol. Biol..

[63] Takasu Y., Hata T., Uchino K., Zhan Q. (2010). Identification of Ser2 proteins as major sericin components in the non-cocoon silk of *Bombyx mori*. Insect Biochem. Mol. Biol..

[64] Kimura K., Oyama F., Ueda H., Mizuno S., Shimura K. (1985). Molecular cloning of the fibroin light chain complementary DNA and its use in the study of the expression of the light chain gene in the posterior silk gland of *Bombyx mori*. Experientia.

[65] Maekawa H., Suzuki Y. (1980). Repeated turn-off and turn-on of fibroin gene transcription during silk gland development of *Bombyx mori*. Dev. Biol..

[66] Suzuki Y., Giza P.E. (1976). Accentuated expression of silk fibroin genes *in vivo* and *in vitro*. J. Mol. Biol..

[67] Suzuki Y., Suzuki E. (1974). Quantitative measurements of fibroin messenger RNA synthesis in the posterior silk gland of normal and mutant *Bombyx mori*. J. Mol. Biol..

[68] Bello B., Horard B., Couble P. (1994). The selective expression of silk-protein encoding genes in *Bombyx mori* silk gland. Bull. Inst. Pasteur..

[69] Couble P., Moine A., Garel A., Prudhomme J.-C. (1983). Developmental variations of a nonfibroin mRNA of *Bombyx mori* silkgland, encoding for a low-molecular-weight silk protein. Dev. Biol..

[70] Couble P., Chevillard M., Moine A., Revel-Chapuis P., Prudhomme J.-C. (1985). Structural organization of the P25 gene of *Bombyx mori* and comparative analysis of its 5′ flanking DNA with that of the fibroin gene. Nucleic Acids Res..

[71] Inoue S., Tanaka K., Arisaka F., Kimura S., Ohtomo K., Mizuno S. (2000). Silk fibroin of *Bombyx mori* is secreted, assembling a high molecular elementary unit consisting of H-chain, L-chain, and P25, with a 6:6:1 molar ratio. J. Biol. Chem..

[72] Garel A., Deleage G., Prudhomme J.-C. (1997). Structure and organization of the *Bombyx mori* sericin 1 gene and of the sericins1 deduced from the sequence of the ser 1B cDNA. Insect Biochem. Mol. Biol..

[73] Zhang Y., Zhao P., Dong Z., Wang D., Guo P., Guo X., Song Q., Zhang W., Xia Q. (2015). Comparative proteome analysis of multi-layer cocoon of the silkworm, *Bombyx mori*. PLoS ONE.

[74] Dhawan S., Gopinathan K.P. (2003). Expression pattern of *Cubitus interruptus* from the mulberry silkworm *Bombyx mori* in late developmental stages. Dev. Genes Evol..

[75] Dhawan S., Gopinathan K.P. (2003). Expression profiling of homeobox genes in silk gland development in the mulberry silkworm *Bombyx mori*. Dev. Genes Evol..

[76] Hui C.-C., Matsuno K., Ueno K., Suzuki Y. (1992). Molecular characterization and silkgland expression pattern of *Bombyx* engrailed and invected genes. Proc. Natl. Acad. Sci. USA.

[77] Shimizu K., Ogawa S., Hino R., Adachi T., Tomita M., Yoshizato K. (2007). Structure and function of 5′-flanking regions of a novel transcription enhancing element with a homeodomain protein-binding motif. Insect Biochem. Mol. Biol..

[78] Hui C.-C., Matsuno K., Suzuki Y. (1990). Fibroin gene promoter contains a cluster of homeodomain binding sites that interact with three silk gland factors. J. Mol. Biol..

[79] Takiya S., Kokubo H., Suzuki Y. (1997). Transcriptional regulatory elements upstream and intron of the fibroin gene bind three specific factors POU-M1, Bm Fkh and FMBP-1. Biochem. J..

[80] Takiya S., Ishikawa T., Ohtsuka K., Nishita Y., Suzuki Y. (2005). Fibroin-modulator-binding protein-1 (FMBP-1) contains a novel DNA-binding domain, repeats of the score and three amino acid peptode (STP), conserved from *Caenorhabditis elegans* to humans. Nucleic Acids Res..

[81] Horard B., Julien E., Nony P., Garel A., Couble P. (1997). Differential binding of the Bombyx silk gland-specific factor SGFB to its target DNA sequence drives posterior-cell-restricted expression. Mol. Cell. Biol..

[82] Durand B., Drevet J., Couble P. (1992). P25 gene regulation in *Bombyx mori* silk gland: Two promoter-binding factors have distinct tissue and developmental specificities. Mol. Cell. Biol..

[83] Julien E., Bordeaux M.-C., Garel A., Couble P. (2002). Fork head alternative binding drives stage-specific gene expression in the silk gland of *Bombyx mori*. Insect Biochem. Mol. Biol..

[84] Mack V., Takiya S., Ohno K., Handa H., Imai T., Suzuki Y. (1995). Silk gland factor-1 involved in the regulation of *Bombyx* sericin-1 gene contain fork head motif. J. Biol. Chem..

[85] Matsuno K., Hui C.-C., Takiya S., Suzuki T., Ueno K., Suzuki Y. (1989). Transcriptional signals and protein binding sites for sericin gene transcription *in vitro*. J. Biol. Chem..

[86] Matsuno K., Takiya S., Hui C.-C., Fukuta M., Ueno K., Suzuki Y. (1990). Transcriptional stimulation via SC site of *Bombyx* sericin-1 gene through an interaction with a DNA binding protein SGF-3. Nucleic Acids Res..

[87] Takiya S., Gazi M., Mach V. (2003). The DNA binding of insect Fork head factor is strongly influenced by the negative cooperation of neighbouring bases. Insect Biochem. Mol. Biol..

[88] Foos N., Maurel-Zaffran C., Mate M.J., Vincentelli R., Hainaut M., Berenger H., Pradel J., Saurin A.J., Ortiz-Lombardia M., Graba Y. (2015). A flexible extension of the *Drosophila* Ultrabithorax homeodomain defines a novel Hox/PBC interaction mode. Structure.

[89] Hudry B., Remacle S., Delfini M.-C., Rezsohazy R., Graba Y., Merabet S. (2012). Hox proteins display a common and ancestral ability to diversify their interaction mode with PBC class cofactors. PLoS Biol..

[90] Hirose S., Suzuki Y. (1988). *In vitro* transcription of eukaryotic genes is affected differently by the degree of DNA supercoiling. Proc. Natl. Acad. Sci. USA.

[91] Suzuki Y., Tsuda M., Takiya S., Hirose S., Suzuki E., Kameda M., Ninaki O. (1986). Tissue-specific transcription enhancement of the fibroin gene characterized by cell-free systems. Proc. Natl. Acad. Sci. USA.

[92] Takiya S., Hui C.-C., Suzuki Y. (1990). A contribution of the core-promoter and its surrounding regions to the preferential transcription of the fibroin gene in posterior silk gland extracts. EMBO J..

[93] Tsuda M., Suzuki Y. (1981). Faithful transcription initiation of fibroin gene in a homologous cell-free system reveals an enhancing effect of 5′ flanking sequence far upstream. Cell.

[94] Tsuda M., Suzuki Y. (1983). Transcription modulation *in vitro* of the fibroin gene exerted by a 200-base-pair region upstream from the “TATA” box. Proc. Natl. Acad. Sci. USA.

[95] Tsuda M., Hirose S., Suzuki Y. (1986). Participation of the upstream region of the fibroin gene in the formation of transcription complex *in vitro*. Mol. Cell. Biol..

[96] Tsujimoto Y., Suzuki Y. (1979). The DNA sequence of Bombyx mori fibroin gene including the 5′ flanking, mRNA coding, entire intervening and fibroin protein coding regions. Cell.

[97] Tsujimoto Y., Suzuki Y. (1984). Natural fibroin genes purified without using cloning procedures from fibroin-producing and nonproducing tissues reveal indistinguishable structure and function. Proc. Natl. Acad. Sci. USA.

[98] Fukuta M., Matsuno K., Hui C.-C., Nagata T., Takiya S., Xu P.-X., Ueno K., Suzuki Y. (1993). Molecular cloning of a POU domain-containing factor involved in the regulation of the Bombyx sericin-1 gene. J. Biol. Chem..

[99] Bello B., Couble P. (1990). Specific expression of a silk-encoding gene of *Bombyx* in the anterior salivary gland of *Drosophila*. Nature.

[100] Curtiss J., Heilig J.S. (1995). Establishment of *Drosophila* imaginal precursor cells controlled by *Arrowhead* gene. Development.

[101] Nony P., Prudhomme J.-C., Couble P. (1995). Regulation of the P25 gene transcription in the silk gland of *Bombyx*. Biol. Cell..

[102] Zhao X.-M., Liu C., Li Q.-Y., Hu W.-B., Zhou M.-T., Zhang Y.-X., Peng Z.-C., Zhao P., Xia Q.-Y. (2014). Basic helix-loop-helix transcription factor Bmsage is involved in regulation of *fibroin-H-chain* gene via interaction with SGF1 in *Bombyx mori*. PLoS ONE.

[103] Xu P.-X., Fukuta M., Takiya S., Matsuno K., Xu X., Suzuki Y. (1994). Promoter of the POU-M1/SGF-3 gene involved in the expression of *Bombyx* silk genes. J. Biol. Chem..

[104] Tolkunova E.N., Fujioka M., Kobayashi M., Deka D., Jaynes J.B. (1998). Two distinct types of repression domain in engrailed: one interacts with the groucho corepressor and is preferentially active on integrated target genes. Mol. Cell. Biol..

[105] Gebelein B., McKay D.J., Mann R.S. (2004). Direct integration of *Hox* and segmentation gene inputs during *Drosophila* development. Nature.

[106] Merabet S., Pradel J., Graba Y. (2005). Getting a molecular grasp on Hox contextual activity. Trends Genet..

[107] Hui C.-C., Suzuki Y., Kikuchi Y., Mizuno S. (1990). Homeodomain binding sites in the 5′ flanking region of the *Bombyx mori* silk fibroin light-chain gene. J. Mol. Biol..

[108] Hui C.-C., Suzuki Y. (1990). Homeodomain binding sites in the promoter region of silk protein genes. Dev. Growth Differ..

[109] Oh S.-K., Scott M.P., Sarnow P. (1992). Homeotic gene *Antennapedia* mRNA contains 5′-noncoding sequences that confer translational initiation by internal ribosome binding. Genes Dev..

[110] Kondrashov N., Pusic A., Stumpf C.R., Shimizu K., Hsieh A.C., Xue S., Ishijima J., Shiroishi T., Barna M. (2011). Ribosome-mediated specificity in Hox mRNA translation and vertebrate tissue patterning. Cell.

[111] Xue S., Tian S., Fujii K., Kladwang W., Das R., Barna M. (2015). RNA regulons in *Hox* 5′ UTRs confer ribosome specificity to gene regulation. Nature.

[112] Kmita M., Duboule D. (2003). Organizing axes in time and space; 25 years of colinear tinkering. Science.

[113] Noordermeer D., Leleu M., Splinter E., Rougemont J., de Laat W., Duboule D. (2011). The dynamic architecture of *Hox* gene clusters. Science.

[114] Zakany J., Kmita M., Duboule D. (2004). A dual role for *Hox* genes in limb anterior-posterior asymmetry. Science.

[115] Horard B., Mange A., Pelisser B., Couble P. (1994). Bombyx gene promoter analysis in transplanted silk gland transformed by particle delivery system. Insect Mol. Biol..

[116] Wang H.-B., Nita M., Iwanaga M., Kawasaki H. (2009). βFTZ-F1 and Broad-complex positively regulate the transcription of the wing cuticle protein gene, *BMWCP5*, in wing discs of *Bombyx mori*. Insect Biochem. Mol. Biol..

[117] Tamura T., Kuwabara N., Uchino K., Kobayashi I., Kanda T. (2007). An improved DNA injection method for silkworm eggs drastically increases the efficiency of producing transgenic silkworms. J. Insect Biotech. Sericol..

[118] Imamura M., Nakai J., Inoue S., Quan G.X., Kanda T., Tamura T. (2003). Targeted gene expression using the *GAL4*/*UAS* system in the silkworm *Bombyx mori*. Genetics.

[119] Tamura T., Thibert C., Royer C., Kanda T., Eppen A., Kamba M., Komoto N., Thomas J.L., Mauchamp B., Chavancy G. (2000). Germline transformation of the silkworm *Bombyx mori L*. using a *piggyBac* transposon-derived vector. Nat. Biotechnol..

[120] Takasu Y., Sajwan S., Damion T., Osanai-Futanashi M., Uchino K., Sezutsu H., Tamura T., Zurovec M. (2013). Efficient TALEN construction for *Bombyx mori* gene targeting. PLoS ONE.

[121] Nakade S., Tsubota T., Sakano Y., Kume S., Sakamoto N., Obara M., Daimon T., Sezutsu H., Yamamoto T., Sakuma T. (2014). Microhomology-mediated end-joining-dependent integration of donor DNA in cells and animals using TALENs and CRISPR/Cas9. Nat. Commun..

[122] Toyama K. (1906). Mendel’s low of heredity as applied to the silkworm crosses. Biol. Zbl..

